# Development of method for evaluating cell hardness and correlation between bacterial spore hardness and durability

**DOI:** 10.1186/1477-3155-10-22

**Published:** 2012-06-07

**Authors:** Koichi Nakanishi, Akinori Kogure, Takenao Fujii, Ryohei Kokawa, Keiji Deuchi

**Affiliations:** 1 Laboratory for Core Technology, Development Kirin Beverage Co, Ltd., Technovillage 3F, 1-17-1 Namamugi, Tsurumi-ku, Yokohama, 230-8628, Japan; 2 Shimadzu Analytical & Measuring Centre, Inc, 380-1 Horiyamashita, Hatano, Kanagawa, 259-1304, Japan; 3 Analytical & Measuring Instrument Division, Shimadzu Corp, 1 Nishinokyo Kuwabara-cho, Nakagyo-ku, Kyoto, 604-8511, Japan

**Keywords:** Scanning probe microscope, Bacillus spp, Spore, Hardness, Young’s modulus

## Abstract

**Background:**

Despite the availability of conventional devices for making single-cell manipulations, determining the hardness of a single cell remains difficult. Here, we consider the cell to be a linear elastic body and apply Young’s modulus (modulus of elasticity), which is defined as the ratio of the repulsive force (stress) in response to the applied strain. In this new method, a scanning probe microscope (SPM) is operated with a cantilever in the “contact-and-push” mode, and the cantilever is applied to the cell surface over a set distance (applied strain).

**Results:**

We determined the hardness of the following bacterial cells: *Escherichia coli*, *Staphylococcus aureus*, *Pseudomonas aeruginosa*, and five *Bacillus* spp. In log phase, these strains had a similar Young’s modulus, but *Bacillus* spp. spores were significantly harder than the corresponding vegetative cells. There was a positive, linear correlation between the hardness of bacterial spores and heat or ultraviolet (UV) resistance.

**Conclusions:**

Using this technique, the hardness of a single vegetative bacterial cell or spore could be determined based on Young’s modulus. As an application of this technique, we demonstrated that the hardness of individual bacterial spores was directly proportional to heat and UV resistance, which are the conventional measures of physical durability. This technique allows the rapid and direct determination of spore durability and provides a valuable and innovative method for the evaluation of physical properties in the field of microbiology.

## Background

It is not easy to measure the physical properties of small cells, such as bacteria or organelles. Surface observations and physical measurements at the molecular and atomic level can be made using analytical instruments, particularly scanning probe microscopes (SPMs) [1]. Nonetheless, with some notable exceptions [2], there are very few methods for making direct physical measurements on individual cells. The physical property hardness is particularly informative of the characteristics of a cell, and much can be known about the cell from the determination of this parameter. Cell hardness is thought to be related to the resistance of bacterial spores to heat and UV. In the fields of food and pharmaceutical manufacturing, current methods for determining heat and UV resistance require viable cell counts involving cell culture and can take from a few days to 2 weeks or more to complete. There is a need for a non-cultivation method that can quickly yield results.

**Table 1:** Young' s modulus for vegetative cells and spores of bacteria

**Bacterial strains**	**Vegetative cells**	**Spores**
*S. aureus*	0.211 ± 0.023	–
*E. coli*	0.165 ± 0.033	–
*P. aeruginosa*	0.199 ± 0.023	–
*G. stearothermophilus*	0.222 ± 0.041	0.811 ± 0.048
*B. coagulans*	0.204 ± 0.028	0.682 ± 0.039
*B. subtilis*	0.215 ± 0.018	0.414 ± 0.021
*B. licheniformis*	0.233 ± 0.034	0.329 ± 0.012
*B. megaterium*	0.196 ± 0.022	0.240 ± 0.059

Number of measurement samples = 10.

Young’s modulus, E (N/m) = Physical stress of cell, σ (nN)/Applied strain of cell, ϵ (nm), where ϵ = 50 nm.

As there is no clear definition for the concept of hardness in biology, it is difficult to consider evaluation methods for the cell hardness. The cantilever used in an SPM is a type of spring. We developed a method using the cantilever probe of a SPM to determine the hardness of a cell, an organelle, or other microscopic specimen. Physical stress induced by contact of the probe with the specimen is used to determine the Young’s modulus as a hardness parameter. Here, we report the evaluation of cell hardness for log phase cells and spores of bacteria and compare it to the heat and UV resistance. The relationship between hardness, given by Young’s modulus, and spore characteristics can be used to evaluate spore durability in sporogenic bacteria.

## Results

### Definition of hardness and measurement positions

**Figure 1:**
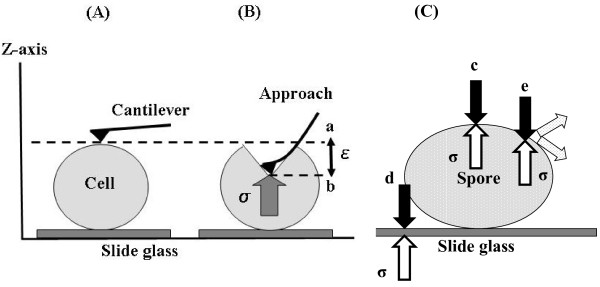
**Principle for measuring cell and spore hardness using the cantilever of the SPM.** (**A**) Position of the cantilever and cell before measurement. (**B**) Cantilever moves a fixed distance along the z-axis (measurement). a: Start position of cantilever, b: Position of cantilever after being depressed to apply strain to the cell, ϵ: Physical strain applied by the cantilever over a fixed distance (50 nm), σ: cell physical stress (nN) (**C**) Diagram of physical stress states for various measurement positions. E: Young's modulus (N/m); σ: cell physical stress (nN); ϵ: physical strain applied by the cantilever (nm) Young’s modulus calculated by E = σ/ϵ. c: Stress in the cell can be accurately measured when the measurement position is the highest point along the x- and y-axes, d: Stress from the slide glass is included in measurements taken at or near the edge of the cell, e: Stress is dispersed if measurement position is slightly away from the highest point.

If hardness is expressed as the amount of work done for a given applied cantilever strain, that amount of work corresponds to a complex three-dimensional movement that would be difficult to measure accurately. However, the error factors are reduced if we limit this movement to the vertical direction along the z-axis, as shown in Figure 1(A) and 1(B) and evaluate Young’s modulus. In this way, the cell can be regarded as a linear-elastic body. The target sample is pressed by the cantilever with a fixed applied strain, *ϵ*, and the repulsive force (stress), σ, (N) is determined (Figure 1(B)). Hooke’s law can be expressed as σ = Eϵ, where E is Young’s modulus (N/m) for log phase cells and spores, and is the physical stress arising in the cell due to the force exerted by the cantilever (depth of compression: m). Hooke’s law holds if the starting point (point a in Figure 1(B)) of the approach curve overlaps with the endpoint of the release curve for the cantilever (point b in Figure 1(B)). Young’s modulus, E, was thus confirmed to be a parameter of cell hardness for various cells and spores (σ/*ϵ*). Young’s modulus of the sample can thus be estimated within the range of the nano-probe evaluation with an identical spring constant.

Figure 1(C) shows a schematic diagram of taking measurements at the center and closer to the periphery of the cell. The SPM scans from the z-axial direction, so evaluations made at the edge cannot be accurately measured. This means that it may not be possible to accurately measure physical stress in the peripheral regions. The diagram shows the forces from the sample when the measurement position is at the apex of both the x- and the y-axis, at a point slightly removed from the apex, and at the edge or at a point near the edge.

**Figure 2:**
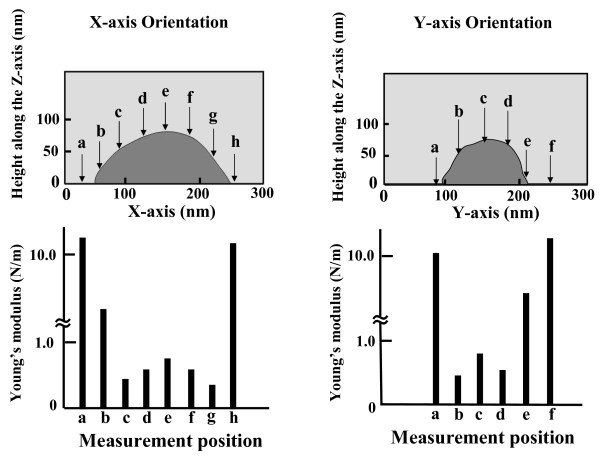
**Representative measurement positions for*G. stearothermophilus*spore and determined Young’s modulus.** Upper slides showed the position to determination of Young’s modulus every 40 nm along the x- and y-axes. Small letters and arrows indicate measurement positions. Lower slides showed the results that Young’s modulus was measured at 40 nm intervals along the x- and y-axes. Letters indicate measurement positions of upper.

The relationship between hardness and point of measurement is shown for a representative spore specimen of *Geobacillus stearothermophilus* NBRC 13737 in Figure 2. Determination of Young’s modulus every 40 nm along the x- and y-axis shows that the most representative values for Young’s modulus are obtained at the point where cell height is greatest along the z-axis. Measurements made at points other than the maximum height are likely to include distributions of physical stress other than those along the z-axis and will not accurately reflect Young’s modulus along the z-axis. Furthermore, if measurements are made near the edge of the specimen, the Young’s modulus measurement is likely to more reflect the hardness of the quartz glass slide than the hardness of the specimen.

**Figure 3:**
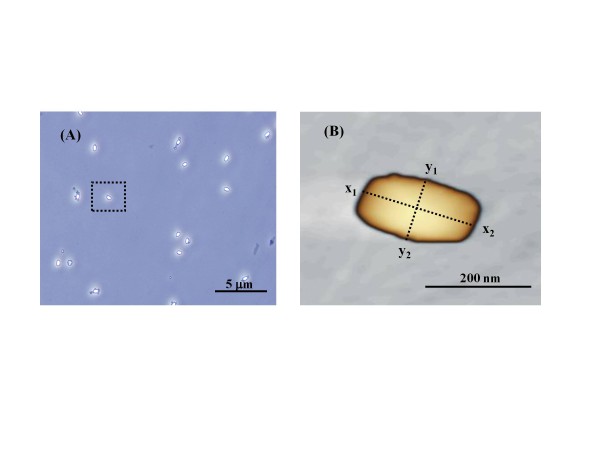
**Measurement position for representative*G. stearothermophilus*strain spore.** (**A**) Phase-contrast microscope image of spores under a phase-contrast microscope to confirm that the spore sample used for measurement has a purity of ≥99%. (**B**) SPM image showing demarcation of x-axis (x_1_ - *x*_2_) and y-axis (y_1_ - y_2_) cross-sections of the spore.

Figure 3 shows the method for determining the measurement position with an SPM. First, *G*. *stearothermophilus* NBRC 13737 was taken as a sample specimen. Spore purity is confirmed to be ≥99% using a phase-contrast microscope, and the spore for measurement is selected (Figure 3(A)). Under a SPM, the measurement position of a single spore is determined as the intersection of the longitudinal x-axis (x_1_ – *x*_2_) and the minor y-axis (y_1_ – y_2_) as shown in Figure 3(B). The point of the maximum height measured on these cross-sections is used to set the indentation and measurement position for the cantilever and was used to determine the representative Young’s modulus for the specimen.

### Young’s modulus of cells and spores

While there were only a small number of samples in the present study, for all bacteria strains tested, hardness of log phase cells was within a narrow range. While there were no clear differences in hardness between the five gram-positive bacteria strains (*S. aureus*, *Geobacillus*, and *Bacillus* spp.) and the two gram-negative strains (*E. coli* and *P. aeruginosa*), the gram-negative bacteria tended to have a lower Young’s modulus. Further experiments using more strains of bacteria are needed to make more detailed examinations of differences between cells in log and stationary phases or for the use of different culture media. Further examination of additional bacteria species is needed to confirm this pattern. Generally, the greater hardness of spores than of vegetative cells for a bacterial strain can be thought of as the basis for the durability of spores. However, while the spores of four *Bacillus* spp. other than *B. megaterium* were harder than the vegetative cells, there was no significant difference in hardness between the spores and vegetative cells for *B. megaterium*. This result may be due to the effect of the exosporium formed by *B. megaterium*, but this will need to be examined in future studies.

### Correlation between young’s modulus and spore durability

**Figure 4:**
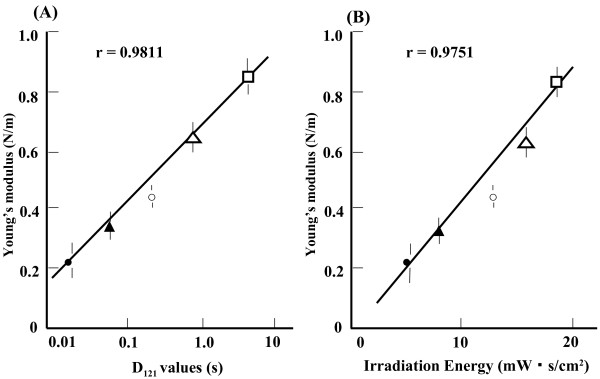
**Correlation between hardness (Young’s modulus) and indirect measurements of heat and UV resistance.** (**A**) Young’s modulus *vs.* D_121_ values. (**B**) Young’s modulus *vs.* irradiation energy. Key for both panels: □, *Geobacillus stearothermophilus*; Δ, *Bacillus coagulans*; ○, *Bacillus subtilis*; ▴, *Bacillus licheniformis*; ●, *Bacillus megaterium.*

The comparison of spore durability and Young’s modulus is shown in Figure 4. A plot of Young’s modulus against an index of heat resistance, D_121_, or the time required to kill 90% of the viable bacteria by heating to 121°C shows a linear relationship (r = 0.9811, Figure 4(A)). Similarly, Young’s modulus plotted against an index of UV resistance, irradiation energy, or the product of irradiation intensity and irradiation time at which 10% of bacteria survive shows a linear relationship (r = 0.9751, Figure 4(B)).

## Discussion

Greater cell hardness for spores than for vegetative cell of sporulating bacteria can be assumed to confer greater physical durability, such as heat resistance to spores. A number of different hypotheses for the formation of more durable spores have been proposed. Setlow [3] attributed the high physical durability of spores primarily to the low water content in the core of the spore, which is due to compression and dehydration during spore cortex formation processes. Porham *et al.*[4] proposed dehydration within the spore core as the major factor. Paredes-Sabja *et al.*[5] hypothesized that minute structures composed of dipicolinic acid (DPA) with a dehydration function in the core of the spore were responsible for the increased durability. Grehardt *et al.*[6] reported that for *Bacillus* spp., lower water content within the spore was correlated with higher durability and that positive correlations were seen between the water content and each thermal processing D value. Taken together, increased hardness resulting from reduced water content in the spore is related to durability.

In particular, hardness is thought to be acquired due to the dehydrating effect of the formation of bonds between dipicolinic acid, which is a unique component of spores, and calcium. The dehydrated spore has a strong and resilient structural hardness that confers strong physical durability. The difference in hardness between vegetative cells and spores of sporulating bacteria and the strong correlation between hardness and heat and UV resistance of the spores support this hypothesis.

Further work is required to expand this method to other applications. A first step to understanding the applicability of this method is to investigate additional spore specimens. While a correlation was found between hardness and durability in the present study, a spore with high cell hardness is not necessarily UV resistant. Nicholson *et al.*[7] noted the relationship between heat resistance and divalent metal ions and the involvement of DNA repair enzymes in UV resistance. These enzymes have a direct relationship to the resistance mechanism, and it is likely that bacteria that form harder spores have higher divalent metal ion concentration and DNA repair enzyme production. The mechanism of spore durability is not yet fully clarified, but the present study offers some new and relevant findings. More heat- and UV-resistant mutants will need to be studied to clarify whether the correlations observed in the present study are direct correlations or whether they indicate indirect agreement. Also, in addition to dehydration of the spore, it is likely that differences in the spore coat contribute to differences in hardness; spore coat deletion variants will make it possible to investigate the correlation between hardness and durability in greater detail. The correlations between spore hardness and heat and UV resistance observed in the present study should be tested using alternative methods for measuring hardness. We expect that this new analytical method can be used to directly measure hardness on any type of cell and will have applications in a wide range of research, including pharmaceutical and food product testing.

## Conclusions

We developed methods to directly measure the physical properties of a single cell using bacterial spores and a SPM. The hardness of a single cell is described in terms of its Young’s modulus. Using this technique, the hardness of bacterial cells could be measured for the first time. We used this technique to measure the hardness of individual spores of spore-forming bacteria strains and observed a high correlation between spore hardness and resistance to heat and UV, which are conventional measures of physical durability. The present technique will permit the rapid measurement of spore durability and will be a powerful tool for clarifying its mechanisms. Most importantly, this technique allows the physical evaluation of a single spore, which may lead to breakthroughs in the field of microbiology.

## Methods

### Bacterial strains

For hardness measurements, five *Bacillus* spp. with different heat resistances and typical gram-positive and gram-negative bacteria were selected from the ThermoKill Database R8100 [8]. Vegetative cells of three strains of “non-sporulating” bacteria were used: *Staphylococcus aureus* subsp. *aureus* NBRC 100910, *Escherichia coli* IFO 3301 and *Pseudomonas aeruginosa* ATCC 10145. Vegetative cells and spores of the following sporulating *Bacillus* spp. and related genera were used: *Geobacillus stearothermophilus* NBRC 13737, *Bacillus coagulans* DSM 1, *B. subtilis* NBRC 13719 T, *B. megaterium* NBRC 15308 T and *B. licheniformis* NBRC 12200.

### Culture and pretreatment methods

All test bacterial strains were cultured in Difco nutrient broth at 35°C, except for *G. stearothermophilus*, which was cultured at 60°C. The vegetative cells were used for experiments after they were confirmed to be in log phase (OD_600_ = 0.8–1.0) at 4 to 12 h after inoculation. Spore-forming *Bacillus* spp. were examined by phase-contrast microscopy to ensure that no spores were present before being used as vegetative cells in experiments. Bacterial log phase cells were prepared for hardness evaluation by centrifuging at 8,000 × *g*, washing the pellet with pure water, mounting cells on quartz slide glass, and drying. Samples were taken prior to the washing step to evaluate the effects of washing with pure water on viable cell count. *Bacillus* spp. spores were collected from 96-h cultures and were prepared for analysis in the same manner as the vegetative cells following resuspension in lysozyme (10 mg/ml) in 10 mM Tris–HCl buffer and centrifugation at 8,000 × *g* to remove vegetative cells. The prepared spore suspensions were examined by phase-contrast microscopy to confirm that ≥99% of the spores were mature before use in experiments.

### Hardness measurement method (young’s modulus)

With the exception of long rod-shaped bacteria, the shape of bacterial cells is close to spheroidal, and the size is in the range of 0.1 to several micrometers (z-axis length of 0.1 to 1 μm). To make the hardness evaluation, assumptions were made that bacterial cells are limited in size and behave as linear-elastic bodies. For hardness determination of each strain, 10 cells of similar shape and size were selected for analysis, and the mean of the measurements was calculated.

The hardness of the cells was evaluated with an SPM equipped with a Nano Search Microscope (SFT-3500, Shimadzu Corporation). The cantilever probe (Olympus, OMCL-AC240TS) was pressed against the surface of a cell with a physical strain assumed to be ϵ = 50 nm, the repulsive force, *σ* (nN) was measured, and these two values were used to calculate Young’s modulus E (N/m) = *σ*/*ϵ*.

Taking into account that measurements are performed on live samples, probes with a light spring constant of k = 2 N/m were used to make hardness measurements. To avoid the effects of interlot variation, each set of comparative tests was carried out with probes from a single lot.

### Heat and UV resistance

D_121_ values, or the time required to kill 90% of viable bacteria with heating to 121°C, for samples cultured in M/15 phosphate buffer (pH 7.0) and heated in thermal death time (TDT) tubes [9] were obtained from the ThermoKiLL Database R8100 [8]. The D_121_ value for each *Bacillus* spp. was used as the heat resistance parameter. For UV resistance, the UV exposure (mW·sec/cm^2^) required to kill 90% of the bacteria was determined. Spore suspensions at 10^5^ CFU/ml in pure water were placed at a depth of 5 mm in a 90 mm Petri dish and irradiated at an intensity of 0.3 mW/cm^2^ using a GLQ lamp (15 W, UVC of mainly 185 nm, Toshiba Lighting & Technology Corporation) placed at a distance of 10 cm. Viable bacteria counts were taken every 10 min for 60 min [10,11].

## Competing interests

The authors declare that they have no competing interests.

## Authors’ contributions

KN designed experiments, analyzed data, and prepared the manuscript. AK, TF, and RK developed and supervised the use of the Nano Search microscope SFT-3500 for microbiological research. KD supervised experiments. All authors discussed the results and commented on the manuscript. All authors read and approved the final manuscript.
